# Optimization and validation of blade parameters for inter-row weeding wheel in paddy fields

**DOI:** 10.3389/fpls.2022.1003471

**Published:** 2022-10-10

**Authors:** Yongzheng Zhang, Liang Tian, Chengmao Cao, Chengliang Zhu, Kuan Qin, Jun Ge

**Affiliations:** ^1^ School of Engineering, Anhui Agricultural University, Hefei, China; ^2^ Quality Supervision Department, Anhui Province Agricultural Machinery Test and Appraisal Station, Hefei, China

**Keywords:** paddy field, weeding wheel, blade angle, optimal composition, field test

## Abstract

The performance of existing rice-paddy weeding machines is not optimal. In this study, the influence of the installation angle of the weeding-wheel blade on cutting resistance and soil-slippage ability was analyzed. The optimal blade angle of the weeding wheel (i.e., the angle at which the resistance to the weeding wheel is minimal and the disturbance speed of the soil maximal) was shown to be< 20°; numerical simulation showed the actual optimal value to be 0°. Different weeding depths (30, 40, and 50 mm), rotation speed of weeding wheel (120, 180, and 240 r/min), and weeder forward speeds (0.3, 0.6, and 0.9 m/s) were used as test factors, and the rates of seedling injury and weeding were used as performance-evaluation criteria to optimize the machine in a secondary orthogonal-rotation combination test. Field experiments showed that the weeding wheel can exhibit optimal working performance under the operating conditions of weeding depth of 39 mm, rotation speed of 175 r/min, and forward speed of 0.6 m/s. The seedling injury and weeding rates were 4.4% and 88.2%, respectively, which were consistent with the numerically predicted results and met the agronomic requirements. This study provides a technical reference for the improvement of paddy-field weeding components.

## 1 Introduction

Weeds spread easily in the rice-field ecosystem and cause great harm to the growth of rice (Zhang et al., 2015; Zhang et al., 2016; Kaur et al., 2018). Weed control in paddy fields is an important part of yield assurance (Armengot et al., 2013; Pannacci and Tei, 2014). Mechanical weeding has attracted wide attention as a non-chemical weed-control method (Shaner and Beckie, 2014). It has the advantages of low labor intensity, high work efficiency, and low cost (Fontanelli et al., 2013); it can also disturb the field soil and increase its oxygen content, promoting the growth of crop roots. Different types of weed control machinery for paddy fields have been developed, among them battery-assisted inter-row weeders (Jiao et al, 2022), self-propelled inter-row weeders (Tang et al., 2021a), and vision-based weeding robots (Wang et al., 2018). However, most of these studies focused on the light simplification and intelligent design of the whole machine rather than on the optimization of the characteristics of key components. Only a few studies have been conducted on the relationship between the structure of key weeding components and weeding performance. In actual operation, weeding machines are prone to problems such as excessive working resistance, low weeding rates, and excessive damage to crop seedlings.

In other branches of agriculture, the influence of the parameters of soil-contacting components on the working resistance and effectiveness has been studied. Fang et al. (2016) approached this problem through numerical simulation, using discrete-element methods. Bentaher et al. (2013) used three-dimensional reconstruction and the finite-element method to simulate the interaction between the plow and the soil, obtaining the working angle of the minimum resistance of the plow. These researchers assumed dryland soil, but a similar approach may be applicable to weeding in paddy fields. The soil in rice fields has a strong adhesive force, and the weeding wheel is subject to great resistance during operation. Discovering the optimal installation angle for the blade of the weeding wheel would reduce the operating resistance and improve the weeding performance.

In this study, the weeding-wheel blade-mounting angle and the field-operation parameters are optimized. The relationship between the installation angle of the weeding-wheel blade and the resistance and weeding performance of the weeding wheel is determined through theoretical analysis and simulation, and the optimal combination of parameters for weeding-wheel operation is determined through orthogonal-rotation tests. The operational performance of the weeding wheel is verified by conducting field experiments.

## 2 Material and methods

### 2.1 Overall structure of weeder

**Figure 1:**
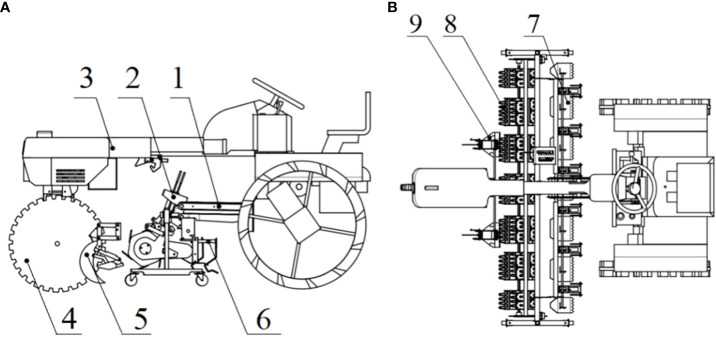
Schematic diagram of a paddy field weeding device: **(A)** top view; **(B)** bottom view. (1. Suspension device. 2. Hoeing-depth-adjusting device. 3. Frame. 4. Walking device. 5. Anti-winding knife. 6. Intra-row weeding device. 7. Mud scraper. 8. Inter-row weeding device. 9. Depth-limit plate.).

The team has developed a paddy-field inter-row weeder (**Figure 1**) that typically contains rake-tooth weeding wheels, a frame, front and rear vehicle wheels, a hoeing-depth-adjustment device, and related accessories (Tian et al., 2022). The most important working part is the inter-row weeding device, installed in front of the driver’s seat between the front and rear wheels of the weeding machine.

**Figure 2:**
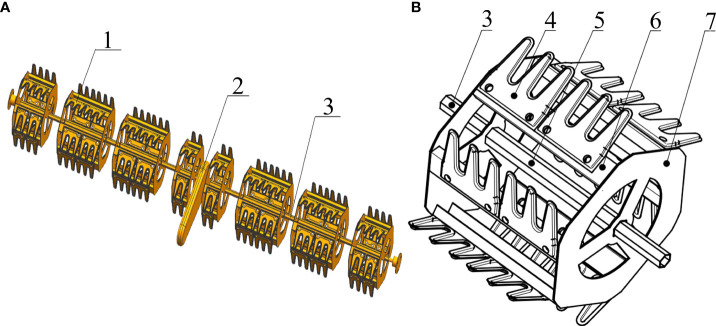
Schematic diagram of **(A)** inter-row weeding component and **(B)** individual weeding wheel (1. Weeding wheel. 2. Gear box. 3. Square shaft. 4. Rake tooth. 5. Bearing sleeve. 6. Mounting base. 7. Side plate.).

The weeding device includes a square shaft, weeding wheels, and a gearbox (**Figure 2A**). Each weeding wheel is composed of a shaft sleeve, blades, blade-mounting seats, and side plates, as shown in **Figure 2B**. The width of a weeding wheel is 220 mm; its radius is designed to be 125 mm. The six blades have rake teeth; the blade length is 120 mm (Tian et al., 2021).

During operation, the weeding wheel rotates. As it enters the soil, each blade produces a downward pressure on the soil and weeds. Subsequently, when the rake tooth is unearthed, the soil and weeds are thrown back; thus, weeds are removed and the soil is loosened.

### 2.2 Optimization range analysis of blade parameters of weeding wheel

**Figure 3:**
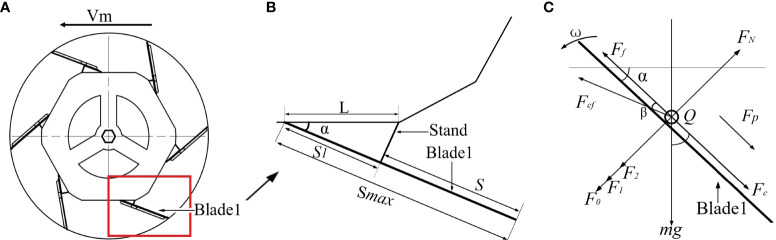
Operation of weeding wheel: **(A)** side view of wheel; **(B)** enlarged view of blade-attachment site, showing the installation angle α; **(C)** soil-particle stress-analysis diagram.


Each blade is installed on the hexagonal wheel at a fixed angle *α*. As shown in **Figure 3A**, stands are installed between the blades and the wheel to ensure stability; thus, α is a constant. In actual operation, the existence of the stands means thathe blades cannot be completely buried. The installation angle (α) of the blade will affect the actual penetration length, and thus the resistance to the weeding wheel during operation. **Figure 3B** reveals that the relationship between *α* and the actual penetration length of the blade is

(1) $$S_{1}=L\cos\alpha \;,$$

(2) $$S=S_{max}-S_{1}=S_{max}-L\cos\alpha \;,$$

where *S*_1_ is the length of blade not buried, *L* is the length of the blade-installation side, *S*_*max*_ is the total length of the blade, and all distances are in mm.

In Eq. (2), when the total length of the blade and the installation-side length are fixed, a smaller *α* corresponds to a smaller *S*. As per an established formula (Liu et al., 2019) and Eq. (2), the resistance of the weeding wheel to cut the soil can be expressed as


(3) $$P_{c}=k_{c}\frac{3Sv_{m}}{9.55\pi }=(S_{max}-L\cos\alpha )\frac{3k_{c}v_{m}}{9.55\pi }\;,$$

where *P*_*c*_  is the resistance of the weeding wheel [N],  *k*_*c*_  is the specific energy consumption in cutting [N·m/mm ^3^], and  *v*_*m*_ is the forward speed of the weeding wheel as it cuts the soil [m/s].

In Eq. (3), *α* is the only variable. It can be seen that *P_c_* decreases when *α* also decreases; when *α* = 0°, the resistance of the blade to cutting the soil is the smallest.

When the blade is out of the soil, any soil wrapped with weed roots that has accumulated on it will slip off under the action of gravity. Different angles of α result in different values of the sliding force, which should be maximized for the best soil-desorption effect. To explore the influence of angle on the soil particle sliding force, mechanical analysis was conducted on soil particles on the blade out of the soil, as shown in **Figure 3C**.

The soil particles are subjected to gravitational force –*mg* , the supporting force of the blade –*F*_*N*_ , and sliding friction *F*_*f*_ . (All forces are measured in newtons.) Decompose *mg* into a component force *F*_0_ perpendicular to the blade downward and a component force parallel to the blade plane reveals that


(4) $$F_{0}=F_{1}+F_{2}=mg\;\text{cos }\alpha$$

where *F*_1_ is the component that provides centripetal force to the soil, and *F*_2_ is the reaction to *F_N_*. The normal force, *F*_*nf*_ , on soil particles can be expressed as


(5) $$F_{nf}=ma_{nv}=F_{N}-F_{2,}$$

where *a*_*nv*_ is the normal relative acceleration of soil particles [m/s^2^]. The normal relative force,  *F*_1_ , of the soil particles can be decomposed into the centripetal force, *F*_*cf*_ , and the tangential component, *F*_*c*_ , of the soil-implicated movement, related by


(6) $$F_{cf}=ma_{ta}=m\omega^{2}r_{Q}=\frac{F_{c}}{\text{cos }\beta },$$

where *a_ta_* is the soil-implicated acceleration [m/s^2^], *ω* is the angular speed of the weeding wheel [rad/s], *r_Q_* is the distance between the soil particle and the rotation center [mm], and *β* is the angle between the soil particles and the rotation center line and the blade plane[°].   The tangential force, *F*_*ta*_ , of the soil particles parallel to the downward direction of the blade is


(7) $$F_{ta}=ma_{tv}=F_{c}+mg\text{sin}\alpha -\mu F_{N},$$

where *a_tv_* is the tangential relative acceleration of the soil particles [m/s^2^] and *μ* is the coefficient of sliding friction between the soil particles and the blade of weeding wheel. According to Eq. (4) – (7), the resultant force *F*_*p*_ of soil particles is


(8) $$F_{p}=ma_{tv}+\mu ma_{ta}sin\beta =mg\text{sin}\alpha -\mu mg\text{cos}\alpha +m\omega^{2}r_{Q}\text{cos}\beta +\mu m\omega^{2}r_{Q}\text{sin}\beta$$

To find the value of *α* that maximizes *F*_*p*_ , perform the following calculation:


(9) $$\{\begin{array}{c} f\left(\alpha ,\beta \right)=F_{p}\\ f{'}_{\alpha }\left(\alpha ,\beta \right)=0\\ f{'}_{\beta }\left(\alpha ,\beta \right)=0 \end{array}$$

It can be drawn by calculating Eq.(9) that when *α* is 20°,  *F*_*p*_ gets the maximum value.

To reduce the cutting resistance as much as possible and ensure the soil removal ability of the blade, the installation angle of the blade should be between 0° (where the resistance is smallest) and 20° (where the soil-removing ability is greatest).

### 2.3 Simulation test

In order to determine the best installation angle of the blade of the weeding wheel, five weeding wheel models were designed with *α* = 0°, 5°, 10°, 15°, 20°, and their operation was simulated and analyzed by EDEM discrete-element modeling software. Because weed growth in paddy fields is complex, directly analyzing a blade–water–soil model using simulation software is difficult. Therefore, in this study, *α* was evaluated from the resistance received by the weeding wheel in the process of operation and from the movement speed of soil particles.

During the weeding of paddy fields, the soil in the tillage layer is saturated with water after irrigation and bubble-field treatment. In this study, two different particle sizes were chosen to represent soil and water in the paddy soil layer. To save simulation time and reduce storage space, the simulation of soil particles was appropriately enlarged. Soil particles with a radius of 5 mm were used to simulate paddy soil, and the shear modulus of water in the simulation experiment was set to 1.0×10^8^ Pa. Based on a review of the paddy-soil literature (Yang et al., 2021), Poisson’s ratio of the paddy soil was taken to be 0.5, the shear modulus to be 1.0×10^8^ Pa, the density to be 1860 kg/m^3^, and the surface energy to be 0.15 J/m^2^. The weeding wheel was made of steel, with Poisson’s ratio 0.3, elastic modulus 7.0 × 10^10^ Pa, and density 7800 kg/m^3^. To meet the requirements of the medium-tillage weeding simulation, a virtual soil tank was established with length × width × height 1000 mm × 450 mm × 100 mm, and a 20-mm-thick water layer was established on the soil tank (Jiang et al., 2020).

**Table 1** lists the contact parameters of the simulation model. To ensure the continuity of the simulation process (Zhang et al., 2020), the fixed time step was set to 4.15×10^6^ s (20% of the Rayleigh time step). The data storage interval was 0.01 s. After the simulation, the results were exported and analyzed using the EDEM software post-processing tool module.

**Table 1:** Contact parameter table of simulation model.

Category	Coefficient of restitution	Coefficient of static friction	Coefficient of kinetic friction
Weeding wheel: soil	0.10	0.20	0.20
Weeding wheel: water	0.05	0.05	0.01
Soil: soil	0.05	0.05	0.05
Soil: water	0.70	0.10	0.05
Water: water	0.01	0.01	0.01

**Figure 4:**
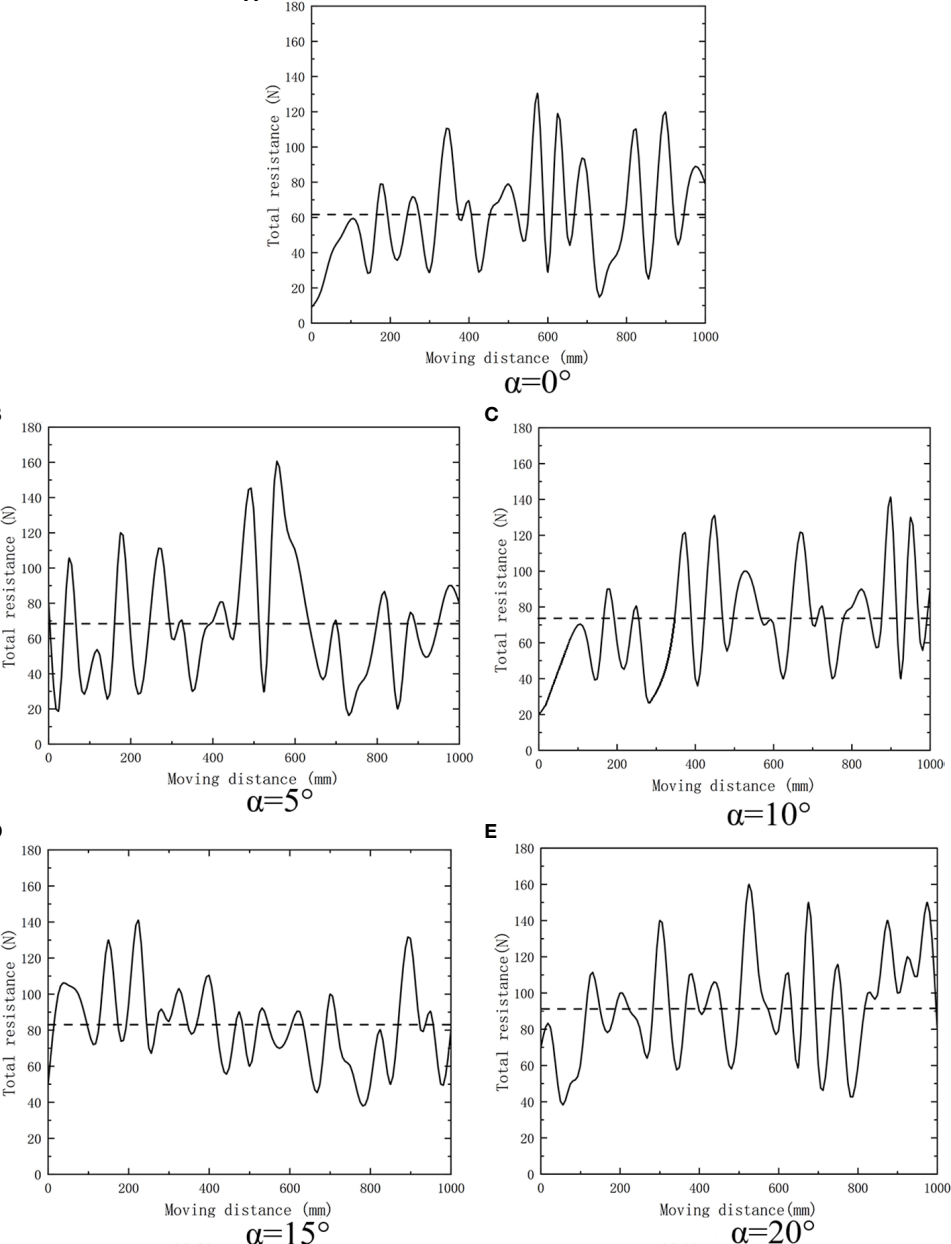
Comprehensive resistance diagram of weeding wheel at five installation angles.

**Figure 4** shows the analyzed comprehensive resistance of the weeding wheels with different blade-installation angles according to the simulation results:

- When *α*=0°, the resistance fluctuated in the range of 40–130 N, and the average resistance was approximately 61 N.
- When *α*=5°, the resistance fluctuated in the range of 10–150 N, and the average resistance was approximately 68 N.
- When *α*=10°, the resistance fluctuated in the range of 20–150 N, and the average resistance was approximately 73 N.
- When *α*=15°, the resistance fluctuated in the range of 40–150 N, and the average resistance was approximately 82 N.
- When *α*=20°, the resistance fluctuated in the range of 40–160 N, and the average resistance was approximately 91 N.

Thus, with the increase in α, the resistance of the weeding wheel increases.

**Figure 5:**
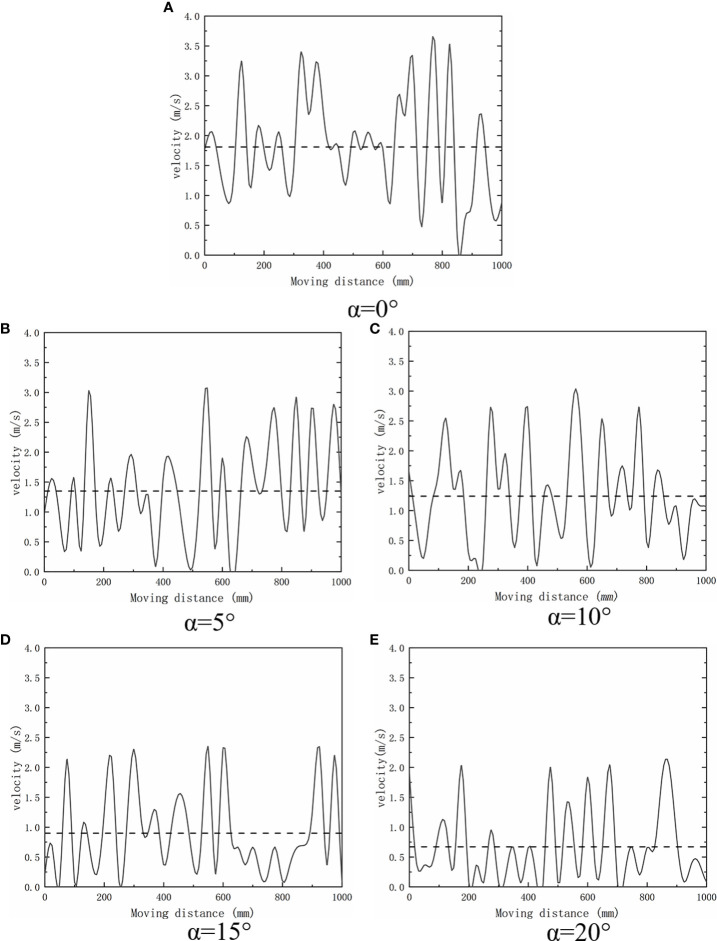
Velocity map of soil particles at five installation angles.

**Figure 5** shows the relationship between soil particle velocity and blade installation angle of weeding wheel.

During the weeding operation, the disturbance of the weeding wheel on the soil can be expressed by the velocity of the soil-particle movement:

- When *α*=0°, the maximum value of soil particle velocity was 3.51 m/s, and the average value was 1.81 m/s.
- When *α*=5°, the maximum value of soil particle velocity was 3.02 m/s, and the average value was 1.35 m/s.
- When *α*=10°, the maximum velocity of soil particles was 2.7 m/s, and the average velocity was 1.24 m/s.
- When *α*=15°, the maximum value of soil particle velocity was 2.33 m/s, and the average value was 0.9 m/s.
- When *α*=20°, the maximum value of soil particle velocity was 2.01 m/s, and the average value was 0.67 m/s.

**Figure 6:**
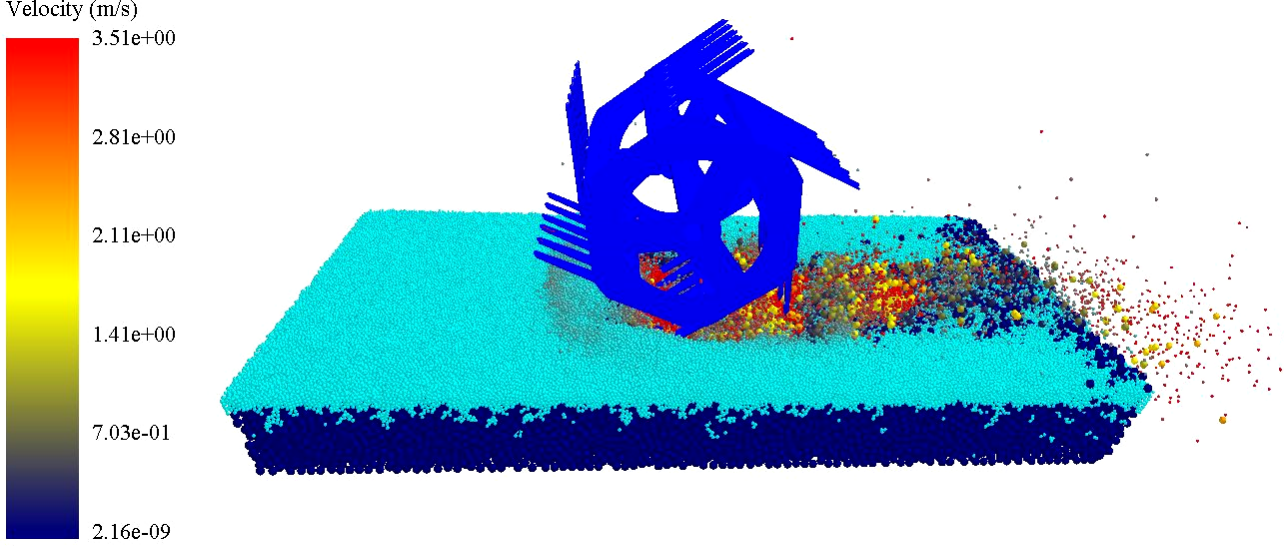
Simulation diagram of soil particle velocity at installation angle α = 0°.

It can be concluded that with the increase of *α*, the velocity of soil particles decreased. As *α* decreased, the resistance of the weeding wheel also decreased and the moving speed of the soil particles increased. When *α*=0°, the average resistance of the weeding wheel reached the minimum value of 61 N, and the movement speed of soil particles reached the maximum value of 3.51 m/s, as shown in **Figure 6**. At this time, the soil disturbance effect was the best. Therefore, the optimal value of *α* was 0°.

When the weeding wheel acts on the rice field, the disturbance of the soil is less when less of the blade has entered the soil. The smaller the reaction force of the soil on the blade of the weeding wheel results in less resistance of the weeding wheel. The cutting pitch of the weeding wheel also decreases, so less soil is cut in a single time, and better soil-breaking takes place. Therefore, when α=0°, the resistance of the weeding wheel is the smallest (Duan et al, 2015). The installation angle of the blade will affect the penetration angle and penetration point (Tang et al., 2021b), which in turn will affect the movement speed of soil particles. In the actual farming environment, if the penetration angle is too large, the backward movement speed of soil particles is reduced, so that the turning speed is lower than the throwing speed, resulting in backwater. Therefore, when α=0°, the penetration angle of the blade is the smallest and the movement speed of soil particles is the largest (Han et al., 2020).

## 3 Results and discussion

### 3.1 Test conditions

**Figure 7:**
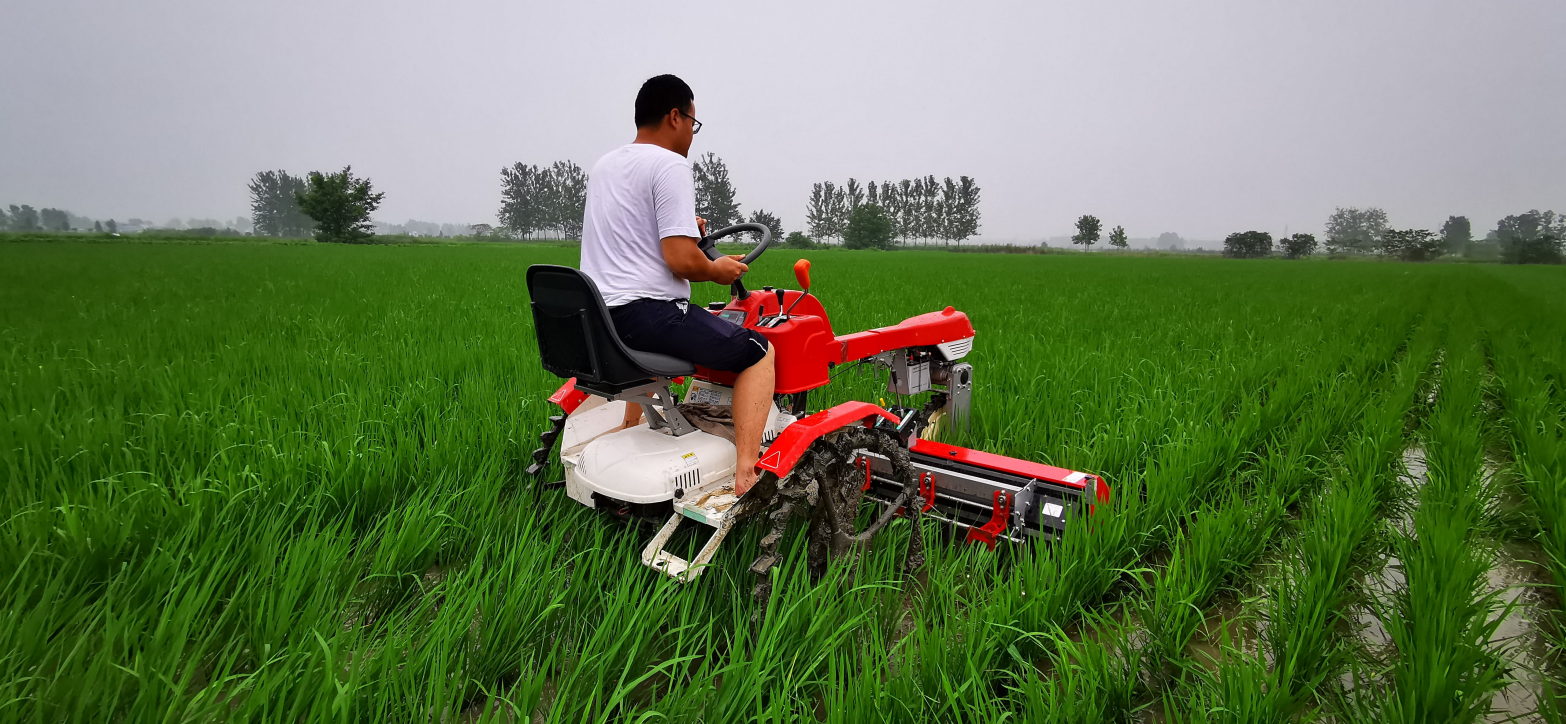
Field-weeding experiment.

To verify the performance of the weeding machine designed in this study, field experiments were conducted in Fenghuang Town, Fengtai County, Huainan City, Anhui Province, China. The experiments were conducted nine days after transplantation, over a test area of ~1.4 hectares. The rice variety cultivated in the experimental field was Nanjing 9108; the average height of seedlings was 255 mm. The rice seedlings grew well, without any obvious diseases or insect pests. No herbicide was applied to the test field. The average height of the weeds was 105 mm; their average root depth was 27 mm. The average density of weeds between rows was approximately 100 plants/m^2^, and the average density of weeds between plants was approximately 25 plants/m^2^. **Figure 7** shows the field-weeding experiment with the paddy-field weeding device.

### 3.2 Test method

Before weeding, the numbers of weeds and seedlings in the test area were determined. After the weeding test was completed, the numbers of weeds removed and not removed were both counted. Each group of data was collected three times, and an average was taken (Jia et al., 2021).

To verify the weeding performance of the rotary rake-tooth paddy-field weeding components designed in this study, the weeding rate (*η*_1_) and damaged-seedling rate (*η*_2_) were selected as the test indexes for field performance test:


(10) $$\eta_{1}=\frac{Z-Z_{1}}{Z}\times 100\%,$$

(11) $$\eta_{2}=\frac{M_{1}}{M}\times 100\%,$$

In Eqs. (10) and (11), *η*_1_ is the percent weeding rate of the row-weeding device; *Z* is the total number of inter-row weeds in rice in the test area; *Z*_1_ is the total number of residual weeds among rice rows after weeding; *η*_2_ is the percent injury rate from weeding between rows; *M*_1_ is the number of damaged seedlings crushed, uprooted, and lodged in the test area after the operation; and *M* is the total number of seedlings in test area.

### 3.3 Experimental design

In the process of weeding, the weeding depth, rotation speed of the weeding wheel, and forward speed also affect the key factors of weeding rate and seedling injury rate. So, weeding depth *A* [mm], rotation speed *B* [r/min], and forward speed *C* [m/s] were used as test factors. Seedling injury rate *R*_1_ and weeding rate *R*_2_ were selected as test indexes. After the test, Design–Expert software was used to process the data, establish the regression equation and the optimization model, and obtain the primary and secondary relationship and the optimal combination of the influence of the test factors on the test indicators. **Table 2** is the design-factor-level coding table.

**Table 2:** Coding with factors and levels.

Canonical variable	Natural variable		
	Weeding DepthA/mm	Rotation SpeedB/r∙min^-1^	Forward SpeedC/m∙s^-1^
–Alpha	30	120	0.3
Lower-level/(-1)	34.22	145.32	0.43
Zero-level/(0)	40	180	0.6
Upper-level/(1)	45.78	241.68	0.77
+Alpha	50	240	0.9

### 3.4 Multi-factor test results and analysis

The results of the quadratic orthogonal-rotation combination test are shown in **Table 3**.

**Table 3:** Protocols and results.

Experimental number	Experimental factor			Experimental index	
	Weeding depthA/mm	Rotation speedB/r min^-1^	Forward speedC/m s^-1^	Seedling injuryRate/%	Weeding rate/%
1	45.78	145.32	0.77	5	86.0
2	50.00	180.00	0.60	5.5	87.5
3	40.00	180.00	0.60	4.1	88.3
4	40.00	180.00	0.60	4.1	88.2
5	45.78	145.32	0.43	5.2	87.1
6	40.00	180.00	0.30	5.1	86.7
7	30.00	180.00	0.60	3.5	86.4
8	34.22	145.32	0.77	5.7	87.7
9	45.78	214.68	0.43	5.4	86.5
10	34.22	214.68	0.43	4.7	86.3
11	45.78	241.68	0.77	5.2	85.3
12	34.22	145.32	0.43	4.7	86.1
13	40.00	180	0.60	4.1	89.1
14	34.22	214.68	0.77	5.6	86.7
15	40	240	0.60	5.4	86.1
16	40	180	0.60	4.1	89.7
17	40	180	0.90	5.3	90.2
15	40	120	0.60	4	86.2
19	40	180	0.60	4.1	89.1
20	40	180	0.60	4.1	89.1

#### 3.4.1 Analysis of variance

F-test and variance analysis were conducted for each coefficient in the regression model. The results of variance analysis for the seedling injury rate *R*_1_ and the weeding rate *R*_2_ are shown in **Table 4**.

**Table 4:** Anova of response surface quadratic model for R_1_ and R_2._.

Sources of Variance	*R_1_*						*R_2_*					
	Sum of squares	Freedom	Mean square	*F*	*P*	Significant	Sum of squares	Freedom	Mean square	*F*	*P*	Significance
Model	6.42	9	0.71	3.28	0.0391	*	30.10	9	3.34	3.92	0.0221	*
*A*	0.91	1	0.91	4.17	0.0684		0.00069	1	0.00069	0.00081	0.9993	
*B*	0.53	1	0.53	2.44	0.1495		0.37	1	0.37	0.43	0.5252	
*C*	0.24	1	0.24	1.11	0.3168		2.43	1	2.43	2.85	0.1225	
*AB*	0.031	1	0.031	0.14	0.7125		0.031	1	0.031	0.037	0.8520	
*AC*	0.66	1	0.66	3.04	0.1117		2.31	1	2.31	2.71	0.1307	
*BC*	0.0012	1	0.0012	0.0057	0.9410		0.21	1	0.21	0.25	0.6294	
*A^2^*	0.69	1	0.69	3.19	0.1045		9.91	1	9.91	11.63	0.0067	*
*B^2^*	1.2	1	1.2	5.54	0.0405	*	17.70	1	17.70	20.76	0.001	**
*C^2^*	3.01	1	3.01	13.85	0.0040	**	1.29	1	1.29	1.52	0.2465	
Residual	2.17	10	0.22				8.53	10	0.85			
Lack of fiit	2.17	5	0.43		0.0613		6.92	5	6.92	4.30	0.0676	
Pure error	0.02	5	0.03				1.61	5	1.61			
Sum	8.59	19					38.63	19	38.63			

P indicates the level of significance of test/. When P is less than 0.01, the test is highly significant, which can be symbolized with “**”; when P is less than 0.05, the test is highly significant, which can be symbolized with “*”.

The data in **Table 4** were subjected to quadratic multiple-regression fitting, and the quadratic-term model was selected to establish the regression model between the seedling injury rate *R*_1_, the weeding rate *R*_2_, and various influencing factors. The following quadratic multiple-regression equations relating *R*_1_ and *R*_2_ to the soil depth *A*, the rotation speed *B* of the weeding wheel and the forward speed *C* are obtained:


(12) $$R_{1}=4.1+0.25A+0.19B+0.13C+0.063AB-0.29AC-0.012BC+0.21A^{2}+0.28B^{2}+0.42C^{2},$$

(13) $$R_{2}=88.91+0.0002A-0.16B+0.41C-0.062AB-0.54AC-0.16BC-0.80A^{2}-1.06B^{2}-0.28C^{2}$$

As listed in **Table 4**, the P-values of the model-misfit terms of the objective functions *R*_1_ and *R*_2_ are 0.0613 and 0.0676, respectively; these are greater than 0.05, indicating no misfit factor. The aforementioned regression equation can be used to replace the real point of the test to analyze the test results.

The analysis of variance in **Table 4** shows that the significant P-values of the *R*_1_ and *R*_2_ models are 0.0391 and 0.0221, respectively; these are less than 0.05, indicating that the model is statistically significant. For objective function *R*_1_, factor *C* ^2^ is very obvious and factor *B* ^2^ is obvious; for objective function *R*_2_, factors *A* ^2^ and *B* ^2^ are very obvious. The F values in **Table 4** indicate the influence of each influencing factor on the test index: larger F values correspond to larger influence. From **Table 3**, experimental factor *A* was the factor exerting the most influence on *R*_1_ and the least on *R*_2_; factor *C* exerted the least influence on *R*_1_ and the most on *R*_2_.

The reason that weeding depth has the greatest impact on the rate of seedling injury is related to the characteristics of rice-root growth. Rice is a typical fibrous root plant (Chai et al., 2019). In the field experiment, when the rice roots were at the tillering stage, the lateral expansion was the largest (Lee et al., 2021); the root group was distributed in a flat oval shape in the range of 20 mm (Zheng et al., 2017; Kahriz and Kahriz, 2018), and the root length was generally between 40 and 60 mm. When the weeding depth is greater than 40 mm, the blade on the weeding roller will inevitably disturb and hurt the roots of rice seedlings (Van et al., 2008). With the movement of the roots, the seedlings will also swing towards the weeding-wheel operation area, leading to further damage. When the deepest depth of the weeding wheel increases, the thickness and area of the soil layer stirred by the weeding wheel also increases. While the weeds are buried and removed, some soil blocks are thrown onto the seedlings, so that the injury rate increases.

The reason that the weeder forward speed has the greatest impact on the weeding rate is that when the rotation speed is fixed, a slower forward speed of the machine implies that a longer time is available for the weeding wheel to work on a given length of soil, increasing the cutting frequency of the blade (Wang et al., 2021) and causing greater soil disturbance (Qi et al., 2015); therefore, the weeding effect is better.

#### 3.4.2 Response surface methodology

**Figure 8:**
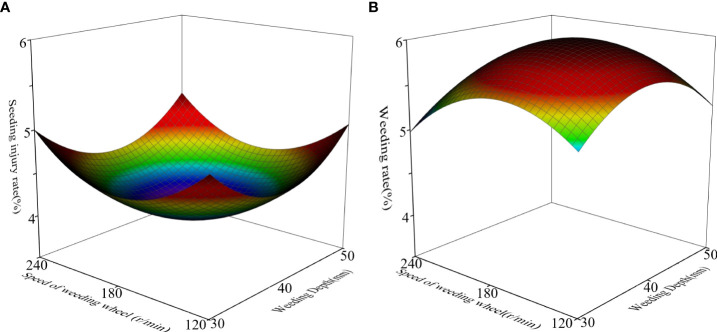
Response surfaces for **(A)** injury rate and **(B)** weeding rate.

According to the response surface generated by the Design-Expert software, for the seedling injury rate, the forward speed of the fixed weeding machine is 0.6 m/s; as shown in **Figure 8A**, when weeding depth *A* is 34.93 mm and rotation speed *B* is 170.89 r/min, the seedling injury rate has the minimum value of 3.9%. For the weeding rate, the forward speed of the fixed weeding machine is 0.6 m/s. As shown in **Figure 8B**, when *A* is 37.76 mm, *B* is 174.67 r/min. The maximum weeding rate is 90.2%.

Because the optimal parameter combination of each test factor of the weeding wheel is different under different indicators, it is impossible to evaluate the optimal parameter combination directly; instead, it is necessary to consider the comprehensive impact of various factors on different indicators (Li et al., 2021). The main purpose of weeding between rows in a paddy field is to eliminate young grass between rows and create favorable conditions for crop growth (Shi et al., 2021), the ideal effect of inter-row weeding is to reduce the seeding injury rate as much as possible while ensuring a high weeding rate. Therefore, the weeding rate index should be given priority. Combining literature results (Colbach et al., 2014) with the actual situation of the field experiment, the optimal parameters of the weeding operation were determined to be *A* = 39 mm, *B* = 175 r/min, *C* = 0.6 m/s. According to the results displayed by the Design-Expert software, in this case, the seedling injury rate is 4%, and the weeding rate is 89%.

### 3.5 Field verification test

On July 5, 2021, a field verification test of the weeding device was conducted in the experimental field described in subsection 3.1, using the optimal parameter combination described in subsection 3.4.2. The seedling injury rate and weeding rate were taken as the test indexes. Five repeated tests were conducted in total, and an average value was taken subsequently. Section 3.2 describes the calculation method for the test structure. **Table 5** lists the processed and analyzed results.

**Table 5:** Field experiment results.

Test number	Seedling injury rate (%)	Weeding rate (%)
1	3.9	88.5
2	5.3	87.4
3	4.2	89.9
4	5.7	87.7
5	3.0	87.5
Average	4.4	88.2

Note that in **Table 5** that the weeding rate and seedling injury rate obtained from the verification test are 88.2% and 4.4%, respectively, very close to the software predictions (89% and 4%). This indicates that the software optimization parameters were accurate and feasible. The weeding quality of the machine under the optimal parameter combination was clearly quite good.

## 4 Conclusion

Existing rice-weeding machines encounter large operating resistances in paddy fields; they have low weeding rates and high seedling-injury rates. To solve these problems, the parameters of the weeding-wheel blades should be optimized. In this study, the key components of mechanical weeding in a paddy field were analyzed. The results showed that when the blade installation angle was 0°, the blade cutting resistance was the smallest; when the blade installation angle was 20°, the blade had the strongest soil-removal ability. Therefore, the range of the installation angle of the weeding-wheel blade should be 0–20°.

EDEM, a discrete-element software, was used to construct a fluid–solid-coupling simulation model of the components and water–soil. The installation angle of the blade of the weeding wheel was taken as the test factor, and the resistance of the weeding wheel and the velocity of soil particles were taken as the test indexes. The test results showed that when the blade installation angle was 0°, the resistance of the weeding roller was the smallest and the velocity of soil particles was the largest. The average resistance was 61 N and the average velocity of soil-particle movement was 1.81 m/s. Therefore, the optimal installation angle of the weeding wheel blade was determined to be 0°.

The combination of machine operation parameters was optimized by conducting a quadratic orthogonal-rotation combination test. The results revealed that the optimal weeding depth was 39 mm, optimal rotation speed was 175 r/min, and optimal forward speed of the machine was 0.6 m/s. The field verification test showed that, for this combination of parameters, the weeding rate was 88.2% and the seedling injury rate was 4.4%, meeting the design requirements of the rice-weeding device.

In this study, the installation angle of the blade of the weeding wheel was optimized, and the effects of the three other key factors (weeding depth, rotation speed, and forward speed) on the weeding and seedling injury rates were studied. However, in actual operation, many other factors (such as the stability of the forward direction of the machine and the cutting effect of the blade on the weed-root system) will affect the weeding rate and the seedling injury rate. Therefore, further research on the effect of the working parameters of key weeding components on weeding-operation quality is needed.

## Data availability statement

The original contributions presented in the study are included in the article/supplementary material. Further inquiries can be directed to the corresponding author.

## Ethics statement

Written informed consent was obtained from the individual(s) for the publication of any potentially identifiable images or data included in this article.

## Author contributions

KQ design the project and performed the literature research. YZ and LT acquired the main data, performed the statistical analysis, and edited the manuscript. CC and JG participated in research and analyzed simulation. CZ participated in research and analyzed the relevant mechanical data and edited the manuscript revision. All authors contributed to the article and approved the submitted version.

## Funding

This work supported by the National Natural Science Foundation of China [grant numbers 52105239]; the Open Fund of State Key Laboratory of Tea Plant Biology and Utilization [grant number SKLTOF20210121]. the Anhui Province Science Foundation of China [grant numbers 2008085QE270].The Anhui Agricultural University Graduate Education Teaching Quality Engineering Project[grant number 2021YJSJY06].

## Conflict of interest

The authors declare that the research was conducted in the absence of any commercial or financial relationships that could be construed as a potential conflict of interest.

## Publisher’s note

All claims expressed in this article are solely those of the authors and do not necessarily represent those of their affiliated organizations, or those of the publisher, the editors and the reviewers. Any product that may be evaluated in this article, or claim that may be made by its manufacturer, is not guaranteed or endorsed by the publisher.

## References

[B1] Armengot L. Jose-Maria L. Chamorro L. Sans F. (2013). Weed harrowing in organically grown cereal crops avoids yield losses without reducing weed diversity. Agron. Sustain. Dev. 33 (2), 405–411. doi: 10.1007/s13593-012-0107-8

[B2] Bentaher H. Ibrahmi A. Hamza E. Hamza E. Hbaieb M. Kantchev G.. (2013). Finite element simulation of moldboard–soil interaction. Soil. Till. Res. 134, 11–16. doi: 10.1016/j.still.2013.07.002

[B3] Chai H. H. Chen F. Zhang S. J. Li Y. D. Liu Z. S. Kang,. Y. J.. (2019). Multi-chamber petaloid root-growth chip for the non-destructive study of the development and physiology of the fibrous root system of oryza sativa. Lab. .Chip 19 (14), 2383–2393. doi: 10.1039/c9lc00396g 31187104

[B4] Colbach N. Biju-Duval L. Gardarin,. A. Granger S. Guyot S. H. M. Meziere,. D.. (2014). The role of models for multicriteria evaluation and multiobjective design of cropping systems for managing weeds. Weed. Res. 54 (6), 541–555. doi: 10.1111/wre.12112

[B5] Duan G. Q. Chen H. T. Li A. Feng Y. N. Yang J. L. Ji W. Y. (2015). Effect of rotation direction of knife teeth configuration on clearing straw unit performance for no-tillage and straw mulching precision seeder. Trans. CSAE. 31 (12), 48–56. doi: 10.11975/j.issn.1002-6819.2015.12.007

[B6] Fang H. M. Ji C. ,. Y. Farman,. A. C. Guo J. Zhang Q. Y. Chaudhry A. (2016). Analysis of soil dynamic behavior during rotary tillage based on distinct element method. Trans. CSAM 47 (3), 22–28. doi: 10.6041/j.issn.1000-1298.2016.03.004

[B7] Fontanelli M. Raffaelli M. Martelloni L. Frasconi C. Ginanni M. Peruzzi A. (2013). The influence of non-living mulch, mechanical and thermal treatments on weed population and yield of rainfed fresh-market tomato (Solanum lycopersicum l.). Span. J. Agric. Res. 11 (3), 593–602. doi: 10.5424/sjar/2013113-3394

[B8] Han B. G Chang G. Y Gao L. Y. Liu Q. Sun S. Dong X. W. (2020). Design and experiment of soybean intra-row weeding monomer mechanism and key components. Trans. CSAM 51 (06), 112–121. doi: 10.6041/j.issn.1000-1298.2020.06.012

[B9] Jia H. L. Gu B. L. Ma Z. Y. Liu H. L. Wang G. Li M. W.. (2021). Optimized design and experiment of spiral-type intra-row weeding actuator for maize (Zea mays l.) planting. Int. J. Agr. Biol. Eng. 14 (6), 54–60. doi: 10.25165/j.ijabe.20211406.6542

[B10] Jiang M. J. Zhang A. Shen Z. F. (2020). Granular soils: from DEM simulation to constitutive modeling. Acta Geotech 15 (7), 1723–1744. doi: 10.1007/s11440-020-00951-7

[B11] Jiao J. K. Wang Z. M. Luo H. W. Chen,. G. L. Liu H. L. Guan J. J.. (2022). Development of a mechanical weeder and experiment on the growth, yield and quality of rice. Int. J. Agr. Biol. Eng. 15 (3), 92–99. doi: 10.25165/j.ijabe.20221503.6978

[B12] Kahriz M. P. Kahriz P. P. (2018). Improving seedling growth and tillering with osmotic priming treatments in rice cv. hamzadere and osmancik 97. J. Anim. Plant Sci. 28 (1), 92–99.

[B13] Kaur S. Kaur R. Chauhan B. S. (2018). Understanding crop-weed-fertilizer-water interactions and their implications for weed management in agricultural systems. Crop Prot 103, 65–72. doi: 10.1016/j.cropro.2017.09.011

[B14] Lee H. S. Hwang W. H. Jeong J. H. Yang S. Y. Jeong N. J. Lee C. K. (2021). Physiological causes of transplantation shock on rice growth inhibition and delayed heading. Sci. Rep-UK 11 (1), 16818. doi: 10.1038/s41598-021-96009-z PMC8376942 34413345

[B15] Li W. H. Li X. G. Deng J. Z. Wang Y. Guo J. P. (2021). Sentiment based multi-index integrated scoring method to improve the accuracy of recommender system. Expert. Sys. Appl. 179, 115105. doi: 10.1016/j.eswa.2021.115105

[B16] Liu D. W. Xie F. ,. P. Ye Q. Ren S. G. Li X. Liu M. Z. (2019). Analysis and experiment on influencing factors on power of ditching parts for 1K-50 orchard ditching. Trans. CSAE 35 (18), 19–28. doi: 10.11975/j.issn.1002-6819.2019.18.003

[B17] Pannacci E. Tei F. (2014). Effects of mechanical and chemical methods on weed control, weed seed rain and crop yield in maize, sunflower and soyabean. Crop Prot 64, 51–59. doi: 10.1016/j.cropro.2014.06.001

[B18] Qi L. Liang Z. W. Ma X. Tan Y. X. Jiang L. K. (2015). Validation and analysis of fluid-structure interaction between rotary harrow weeding roll and paddy soil. Trans. CSAE 31 (5), 29–37. doi: 10.3969/j.issn.1002-6819.2015.05.005

[B19] Shaner D. L. Beckie H. (2014). The future for weed control and technology. Pest. Manage. Sci. 70 (9), 1329–1339. doi: 10.1002/ps.3706 24339388

[B20] Shi Y. J. Xi X. B. Gan H. Shan X. Zhang Y. F. Shen H.. (2021). Design and experiment of row-controlled fertilizing-weeding machine for rice cultivation. Agriculture 11 (6), 527. doi: 10.3390/agriculture11060527

[B21] Tang H. Jiang Y. M. Wang J. W. Guan R. Zhou W. Q. (2021b). Bionic design and parameter optimization of rotating and fixed stem- and leaf-cutting devices for carrot combine harvesters. Math. Probl. Eng 2021 (Pt.4), 8873965.1–8873965.14. doi: 10.1155/2021/8873965

[B22] Tang H. Xu C. S. Wang Q. Zhou W. Wang J. Xu Y.. (2021a). Analysis of the mechanism and performance optimization of burying weeding with a self-propelled inter RowWeeder for paddy field environments. Appl. Sci. 11 (21), 9798. doi: 10.3390/app11219798

[B23] Tian,. L. Cao C. M. Qin K. Fang L. F. Ge J. (2021). Design and test of post-seat weeding machine for paddy. Int. J. Agric. Biol. Eng. 14 (3), 112–122. doi: 10.25165/j.ijabe.20211403.5936

[B24] Tian,. L. Cao C. M. Qin K. Ge J. Fang L. F. (2022). Design and experiment of self-propelled system for paddy field weeder based on the interaction mechanism of wheel-soil. Eng. Agr 42, 1. doi: 10.1590/1809-4430-Eng.Agric.v42n1e20210095/2022

[B25] Van L. S. Mouazen A. M. Anthonis J. Ramon H. Saeys W. (2008). Infrared laser sensor for depth measurement to improve depth control in intra-row mechanical weeding. Biosys. Eng. 100 (3), 309–320. doi: 10.1016/j.biosyst-emseng.2008.03.010

[B26] Wang J. Li X. Ma X. Zhou W. Tang H. (2018). Small tracked and remote-controlled multifunctional platform for paddy field. Int. Agric. Eng. J. 27, 172–182.

[B27] Wang S. Su D. B. Wang Z. M. Jiang Y. Y. Zhang L. N. Tan Y. (2021). Design and experiments of the cam swing rod intra-row weeding device for lettuce farm. Trans. CSAE 37 (21), 34–44. doi: 10.11975/j.issn.1002-6819.2021.21.005

[B28] Yang X. L. Liu G. Li Y. Gao S. H. (2021). Structural optimization of reciprocating seal with magnetic fluid based on orthogonal test design. J. Magn. 26 (2), 229–237. doi: 10.4283/JMAG.2021.26.2.229

[B29] Zhang S. Z. Li Y. H. Kong C. H. Xu X. H. (2016). Interference of allelopathic wheat with different weeds. Pest. Manage. Sci. 72 (1), 172–178. doi: 10.1002/ps.3985 25641926

[B30] Zhang S. Tekeste M. Z. Li Y. Gaul A. Zhu D. Q. Liao J. (2020). Scaled-up rice grain modelling for DEM calibration and the validation of hopper flow. Biosys. Eng. 194, 196–212. doi: 10.1016/j.biosystemseng.2020.03.018

[B31] Zhang J. H. Yao F. M. Hao C. Boken V. (2015). Impacts of temperature on rice yields of different rice cultivation systems in southern China over the past 40 years. Phys. Chem. Earth 87-88, 153–159. doi: 10.1016/j.Pce.2015.08.013

[B32] Zheng L. Luo X. W. Zeng S. Wang Z. M. Liu C. B. QI X. Y. (2017). Shear characteristics of rice root-soil composite. T. CSAM. 48(05), 63–71. doi: 10.6041/j.Issn.1000-1298.2017.05.007

